# Induction of stigma-like structures in saffron (*Crocus sativus* L.): Exploring factors and metabolite analysis

**DOI:** 10.1371/journal.pone.0317186

**Published:** 2025-01-13

**Authors:** Parvaneh Mahmoudi, Ahmad Moieni, Mojtaba Khayam Nekouei, Mohsen Mardi, Ghasem Hosseini Salekdeh

**Affiliations:** 1 Department of Plant Genetics and Breeding, Faculty of Agriculture, Tarbiat Modares University, Tehran, Iran; 2 Department of Systems and Synthetic Biology, Agricultural Biotechnology Research Institute of Iran, Agricultural Research, Education and Extension Organization (AREEO), Karaj, Iran; 3 Faculty of Biological Sciences, Tarbiat Modares University, Tehran, Iran; 4 School of Natural Sciences, Macquarie University, Macquarie Park, Sydney, Australia; Shahrekord University of Medical Science, ISLAMIC REPUBLIC OF IRAN

## Abstract

Saffron (*Crocus sativus* L.) has held significant cultural and medicinal value since the Greek-Minoan civilization. As a triploid spice with vegetative propagation from the Iridaceae family, the three-branch style of *C*. *sativus* flowers, known as saffron, constitutes the most economically valuable part of the plant, renowned for its diverse medicinal properties. This study explores the *in vitro* induction of stigma-like structures (SLSs) from various explants of the Ghaen ecotype flower. The study found that the optimal sampling time for the majority of explants was the third week of October. Ovary explants exhibiting a prolonged response to hormonal treatments for the production of SLSs. Furthermore, intact, and injury ovary explants were found to be the most effective explant types for inducing SLSs. The explants were cultured on MS, 1/2MS, LS and B5 basal media supplemented with various combinations and concentrations of plant growth regulators. The results indicated that the B5 medium, enriched with 5–10 mg/ L BAP and 5–10 mg/ L NAA was the most effective treatment for inducing SLSs in all types of explants. Quantitative and qualitative analyses of saffron compounds in SLSs indicated similarities with natural saffron, albeit at significant lower concentrations: crocin (up to 10.2 mg/g), picrocrocin (up to 4.8 mg/g), and safranal (up to 9.7 mg/g). The highest accumulation of the three studied secondary metabolites was observed in the SLSs of style (24.4 mg/g), stigma (28.3 mg/g), and ovary (21.4 mg/g) explants, respectively. This study introduces a comprehensive procedure for producing SLSs containing the three most important metabolites of saffron for the first time.

## Introduction

**Fig 1:**
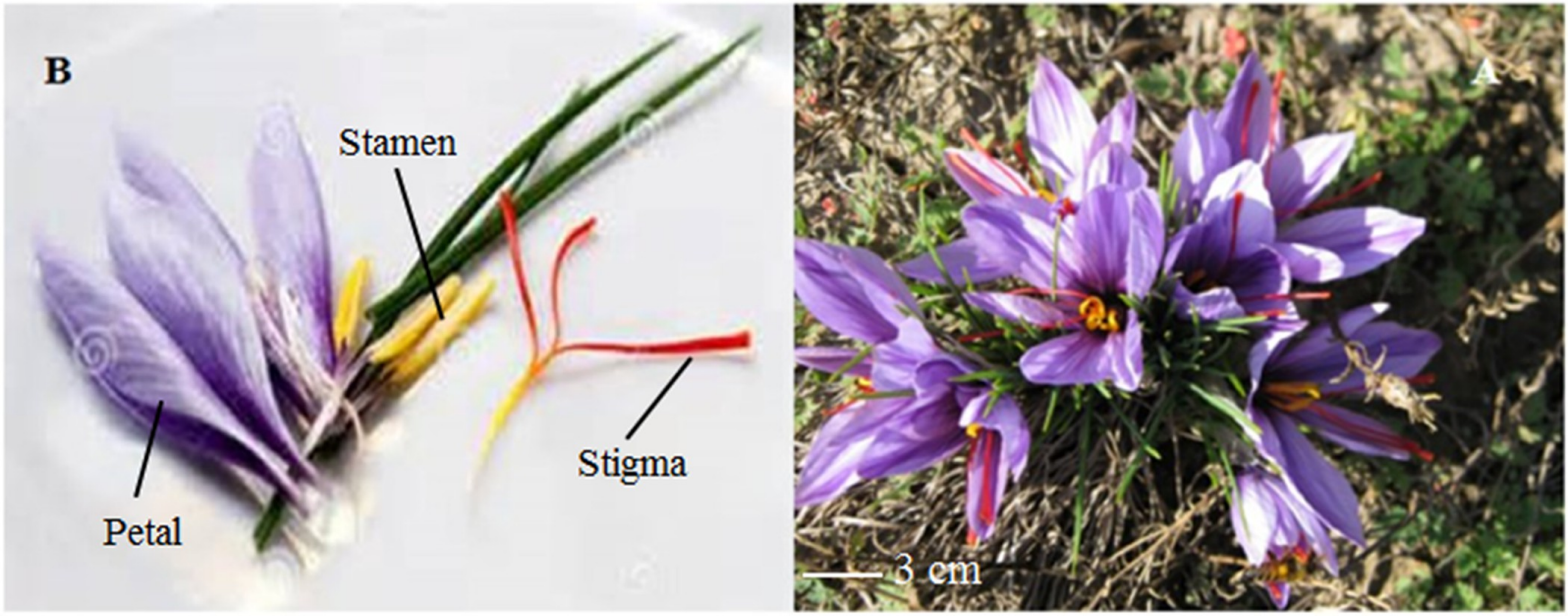
Saffron flower. A) Mature flower, B) different parts of flower; stigmas, stamens, and petals [8].

Saffron (*Crocus Sativus* L.), stands out as the most significant and costly spice plant globally, representing the flagship species within the *Crocus* genus and Iridaceae family. This sterile triploid plant naturally reproduces vegetatively, by formation of daughter corms on a mother corm [1]. The crimson stigmas of *C*. *sativus*, referred to as Saffron, serve as repositories for plethora of valuable and unique components, including carotenoids and apocarotenoids, renowned for their therapeutic effects since the Greek-Minoan civilization [2–4]. Historically, saffron’s essential secondary metabolites, have rendered it a remedy for various human ailments, such as coughs, digestive dysfunctions, colic, insomnia, chronic uterine bleeding, scarlet fever, smallpox, colds, and asthma [5]. Recent investigations have explored its potential therapeutic effects on modern-world diseases like cancer and AIDS [6]. The key metabolites of saffron, namely crocin (C_44_H_64_O_24_), picrocrocin (C_16_H_26_O_7_), and safranal (C_10_H_14_O) impart its color, taste and aroma, respectively. Saffron is a water-efficient perennial herbaceous plant that remains dormant in the corm form for an extended period. Corms become active and initiate meiotic activity at the end of summer, with flowering commencing in autumn (October) after the summer heat subsides. The plant relies solely on vegetative reproduction, where buds on the corms give rise to the next generation, ensuring saffron’s survival. During the flowering season, the flowers precede the appearance of leaves. Each flower features three stamen, five petals, a triple-carpel ovary, three sepals, and three red stigma branches (Fig 1). The number of flowers per corm ranges from one to three, with mature forms exhibiting a 7–10 cm style length culminating in a three branches stigma. Each Saffron stigma weighs approximately 2 mg. To produce 1 kg of dried saffron, around 150 thousand flowers with a wet weight of 70 kg are required, necessitating about 2 to 3 thousand square meters of cultivated land to accommodate this floral abundance [7].

Total world saffron production is estimated to be around 418,000 tons, with approximately 90% originating from Iran and the remaining 10% from countries such as, Afghanistan, Greece, Morocco, Spain and Italy [9]. The trade of saffron has experiences significant growth in recent years due to increased demand in the food, pharmaceutical, and hygiene industries. Consequently, there is a growing emphasis on enhancing stigma production with improved quality. Large scale cultivation of saffron faces various challenges, necessitating of new cultivation methods and chemical techniques for quality assessment and impurity prevention [10].

Additionally, saffron corms are susceptible to various bacterial, fungal and viral pathogens, which can persist even when the corms are removed from the soil for replanting. Despite employing hygienic practices, these pathogens commonly lead to necrosis in leaves, roots and corms, subsequently reducing flowering [11]. The *in vitro* production of SLSs, the economically significant organ of saffron, offers advantages such as reduced production time and the elimination of field-related requirements and certain saffron production processes. *In vitro* production of SLSs is achievable using suitable culture media, and in some cases, the crocin pigments and picrocrocin may exhibit similarity or lighter shades compared to natural stigmas [12].

Numerous studies have explored the *in vitro* formation of SLSs. The investigation was initiated by Himeno and Sano have started in 1987 and has been since pursued by various researchers to optimize the *in vitro* formation of SLSs [11–20]. These investigations have revealed that all parts of the ovary possess the ability to induce stigma formation. Additionally, the formation of SLSs is often linked to the conversion of amyloplasts to chromoplasts and the accumulation of carotenoids [20, 21].

While most studies have focused on SLSs production from different flower organs, a few have identified the most suitable explant concerning both physiological age and the sampling time for SLSs production. Furthermore, there is no clear guidance on achieving the highest efficiency in SLSs production across various flower parts. Due to factors such as the slow growth rate of saffron, vulnerability to viral and bacterial contamination, labor-intensive picking, and substantial manpower requirements, exploring alternative methods for faster and easier saffron production and processing, is imperative. The feasibility may be realized through more comprehensive studies on SLSs production. Accordingly, this study aims to investigate: 1. The effects of different flower organs and their physiological age (sampling time) on SLSs induction and production, 2. The effects of different hormone treatments and culture media on SLSs induction in each type of explant, and 3. Quantity and quality of three secondary metabolites in SLSs. The research aims to provide the first report on determining the optimal sampling time and hormone combination for various explants to produce the highest number of SLSs (through direct and indirect regeneration). Additionally, it examines and compares the ploidy level and the amount of important secondary metabolites in *in vitro* SLSs and field stigmas for the first time.

## Material and methods

### Plant material and surface sterilization

Saffron corms of the Ghaen ecotype, bearing blossomed floral buds, were systematically collected three times per week over 12 weeks, spanning from mid-August to mid-November, from the experimental field of the Agricultural Biotechnology Research Institute of Iran (ABRII).

To minimize the time between collection and laboratory transfer, the corms were swiftly transported. Following the removal of the outer layer, the corms underwent thorough washing with tap water and hand wash liquid for 15 minutes. Given the sensitivity of flower parts, traditional long-term sterilization methods were impractical. In all treatments, following washing with tap water and hand wash liquid, the samples were incubated at 28°C for 24 hours to eliminate possible viral infection. Then, the corms were underwent surface sterilization, involving immersion in 70% ethanol, sodium hypochlorite, and nano-silver solution [22] for different durations (Table 1). Subsequent to each step, the corms were briefly soaked in sterile distilled water, with a final stage involving placement on sterilized filter paper for drying. Then, the buds were dissected from the corms. The flower parts including stigma (red, orange and white base on sampling time), intact ovary, injured ovary (incurred via scalpel), style and the stamens were utilized as explants. Ten days after plant material sterilization, the effectiveness of the treatments was assessed by measuring the percentage of uninfected explants and the number of surviving explants.

**Table 1:** Applied surface sterilization treatments on unopened saffron flower buds.

Treatment No.	Alcohol (S)	Sodium hypochlorite (S)	Nano silver solution (min)	Percentage of healthy samples	Percentage of surviving samples
1	60	900	20	95	21
2	45	900	20	95	23
3	45	600	20	90	28
4	30	600	20	83	37
5	30	300	20	80	35
6	20	300	20	83	39
7	20	60	10	79	38
8	10	60	10	81	52
9	10	30	10	75	61
10	10	10	10	78	60
11	5	10	10	77	75
12	5	5	10	71	78

### Media composition and culture conditions

The explants were subjected to culture on Murashige and Skoog (MS), 1/2MS [23], Linsmaier and Skoog (LS) [24] and B5 medium (B5) [25] basal media, supplemented with various combinations and concentrations of plant growth regulators (cytokinine and auxin) provided from Sigma-Aldrich, USA. All media included 30 g/L sucrose and were solidified with 3 g/L phytagel. Before autoclaving, the pH of all media was adjusted to 5.7–5.8 using HCl/NaOH. The culture media and tools were autoclaved at 121°C for 20, and 30 minutes, respectively. The media were dispensed into disposable Petri dishes (60 × 15 mm) [26].

Sterile explants were placed in Petri dishes, with three explants per dish. The petri dishes were sealed with Parafilm and then placed in a controlled growth chamber at 22°C for inducing SLSs. Subculturing of cultures were occurred at 28-day intervals. Each treatment was replicated three times, and each replication consisted of three Petri dishes.

The initial experiment aimed to identify the optimal sampling time for various flower parts. Experiments were conducted by selecting explants at different physiological ages from mid-August to mid-November. The growth stage of the explants was meticulously monitored under a stereomicroscope. The experiment was carried out based on a factorial experiment in a completely randomized design considering two factors, explant type across six levels (stigma, style, intact ovary, injured ovary, petal, and stamens) and sampling time (third and fourth weeks of August, four weeks in September, four weeks in October and the first two weeks in November). Each treatment was replicated three times, and each replication consisted of three Petri dishes.

In the subsequent year, a second experiment was conducted to assess the impact of plant growth regulators (PGRs) on the induction of SLSs. The experiment utilized the MS medium, supplemented with various combinations and concentrations of commonly used auxins and cytokinines. The auxins included naphthalene acetic acid (NAA), indole-3-butyric acid (IBA), and 2, 4-dichlorophenoxyacetic acid (2, 4-D), and cytokinins included benzylaminopurine (BAP) and kinetin (Kin), resulting in a total of 216 PGRs combinations. The experiment was carried out based on a factorial experiment in a completely randomized design considering two factors, explant type across six levels (stigma, style, intact ovary, injured ovary, petal, and stamens) and PGRs combinations in 216 levels, with 3 replications. The frequency of SLSs formation and the number of SLSs per explant were meticulously recorded approximately 90 days after the initial inoculation, which included three subcultures.

A third experiment was conducted to investigate the influence of culture medium on the induction and formation of SLSs. The media were supplemented with the most effective hormone combinations identified in the second experiment. This experiment employed a factorial design in a completely randomized setup, encompassing two factors: the type of culture medium at four levels (MS, ½ MS, LS, and B5) and the optimal hormone combinations (5 mg/L of NAA and 5 mg/L BAP and 10 mg/L of NAA and 10 mg/L Kin), at 2 levels. The experiment was conducted using injured ovaries as the explants. Each treatment was replicated three times, and each replication consisted of three Petri dishes.

### Statistical analysis

The data obtained from the experiments were subjected to statistical analysis using SPSS Version 18 software [27]. The first experiment’s non-normal data were analyzed using the non-parametric Kruskal–Wallis method. For the second and third experiments, which were conducted based on a factorial experiment employing a Completely Randomized Design (CRD), the data were analyzed with three replications for each scenario.

### Flow cytometry

To assess the ploidy level of the stigma-like structures, flow cytometric analysis was conducted to compare the nuclear DNA content between stigma cells of field plants and *in vitro* stigma-like structures [28]. Each sample, comprising approximately 40–50 mg of stigmas, underwent chopping using a sharp razor blade for approximately 60 s. Subsequently, the homogenate was mixed with 0.5 mL of buffer solution (Nuclei extraction buffer, Partec) and filtered through a 50-μm nylon filter to eliminate large debris. Nuclei were stained using 80 μg mL^−1^ propidium iodide (PI; Fluka, Buchs, Switzerland), and 80 μg mL^−1^ Ribonuclease (RNase) (Sigma, St Louis, MO, USA) was added to the nuclear suspension to prevent the staining of double-stranded RNA. Samples were incubated on ice and analyzed within 10 min. The amount of DNA was determined by examining at least 10,000 cells [29].

Parsley (Petroselinum crispum (Mill.) Fuss) [30], with an approximate nuclear DNA weight of 46.4 picograms, served as an internal standard plant [31]. Fluorescence intensity was measured using a flow cytometer (Partec Gmbh, Munster, Germany) with UV-Laser and High-Pressure Mercury (HBO)-Lamp).

### Metabolite extraction

Field saffron stigmas and *in vitro* SLSs (20 mg) were dried and then suspended in 1 ml of methanol–water (50:50, v/v). The suspension was magnetically stirred for 24 h at 4°C in the dark. After extraction, samples underwent centrifugation at 30,000 ɡ for 35 min to remove plant residues. The supernatant was collected and filtered through a nylon membrane (Acrodisc 13, 0.45 μm pore size, 13 mm diameter, Waters, Milford, MA, USA) [32]. Prior to quantitative chromatographic analysis, 500 μL of 2-nitroaniline (0.5 mg/ml) was added as an internal standard to 500 μL of each tested sample [33].

### Picrocrocin purification

Picrocrocin purification was conducted using preparative High-Performance Liquid Chromatography (HPLC) (Waters Delta Prep 4000). A volume of 500 mL of saffron methanol extract (20 mg/mL 80% Ethanol) was directly injected into a 20 mL/min stream. An HPLC linear gradient was employed, ranging from 90:10 (H_2_O: acetonitrile) to 50:50 over 25 min. Picrocrocin peak elution was monitored at 250 nm. The fraction was concentrated by rotary evaporation, and water was removed through solvent exchange using a C-18 Sep-pack. High-resolution mass spectrometry was employed to confirm both molecular weight and molecular formula [34]. A solution of 1 mg purified picrocrocin in 1 mL of HPLC-grade methanol was prepared. Subsequently, the solution was filtered through a 0.22 μm nylon filter to remove every particulate matter. Then, 5 μL of the picrocrocin solution was injected into the mass spectrometer via the electrospray ionization (ESI) source [35]. The molecular weight and molecular formula of picrocrocin were determined by identifying the peak corresponding to the molecular ion (M+) and analyzing the isotopic pattern of the molecular ion peak, respectively [36]. The collected picrocrocin was determined to be 92% pure [37].

### HPLC

Equal volumes (10 μL) from each replicate were manually injected into the HPLC system (Knauer, pump K-1001) equipped with a C18 column (250 x 4.6 mm, pore size 5 μm; Teknokroma), a pump (Model 600E) and UV–Vis detection. A linear gradient of methanol (50–50% v/v) in water (15% acetonitrile v/v) was employed as a mobile phase, with a flow rate of 1.0 ml min^-1^, and a maximum elution time of 25 min at room temperature [36].

A linear gradient of methanol, ranging from an initial concentration of 50% (v/v) to a final concentration of 50% (v/v) in water, was applied as the mobile phase. The water used in the mobile phase contained 15% acetonitrile (v/v). The gradient was programmed with a flow rate of 1.0 ml min^-1^, and a maximum elution time of 25 min at room temperature.

Two standards, safranal (88%) and crocins (96%), were obtained from Sigma–Aldrich (St. Louis, MO). Picrocrocin was purified as previously described. HPLC-grade methanol (Methanol (MeOH): Ultraviolet (UV) cutoff 205 nm) and HPLC-grade acetonitrile (Acetonitrile (CAN): UV cutoff 190 nm) were supplied by Fisher Chemicals (Pittsburgh, PA).

The quantifications of crocins (440 nm), safranal (310 nm), and picrocrocin (250 nm) were determined on the basis of peak area with comparison to the above-mentioned standards. The internal standard was detected at the above three mentioned wavelengths [32]. Concentrations of crocins, safranal and picrocrocin were measured on a dry weight basis (mg/g). The analyses of each sample were repeated three times.

## Results and discussion

### Plant material surface sterilization

Due to the harsh effects of the sterilization stages on the flower buds, a significant portion of our first-year experiment samples did not survive, primarily due to 1) infections caused by inadequate sterilization in some treatments, and 2) severe sterilization processes in other treatments. To address these challenges, various sterilization treatments were examined to minimize infection and maximize the viability of explants.

The results indicated that a treatment involving a 5-second exposure to 70% ethanol, followed by immersion in a 5% sodium hypochlorite solution containing tween 20 (two drops) for 10 seconds, application of Nano-silver solution for 10 minutes, and a final rinse with sterile distilled water three times proved to be the best and most effective method for surface sterilization of saffron flower buds (Table 1). The duration of sodium hypochlorite treatment was found to have a significant impact on explant contamination. Optimizing the treatment time to 10 seconds was crucial for minimizing contamination while ensuring explant survival. Previous studies have explored various sterilization methods for different parts of the saffron flower [3, 19, 38]. However, this study demonstrated that incorporating a Nano-silver solution into the sterilization process improved effectiveness and led to enhanced samples survival.

### *In vitro* SLSs induction

#### The effect of sampling time on the *in vitro* formation of SLSs

**Fig 2:**
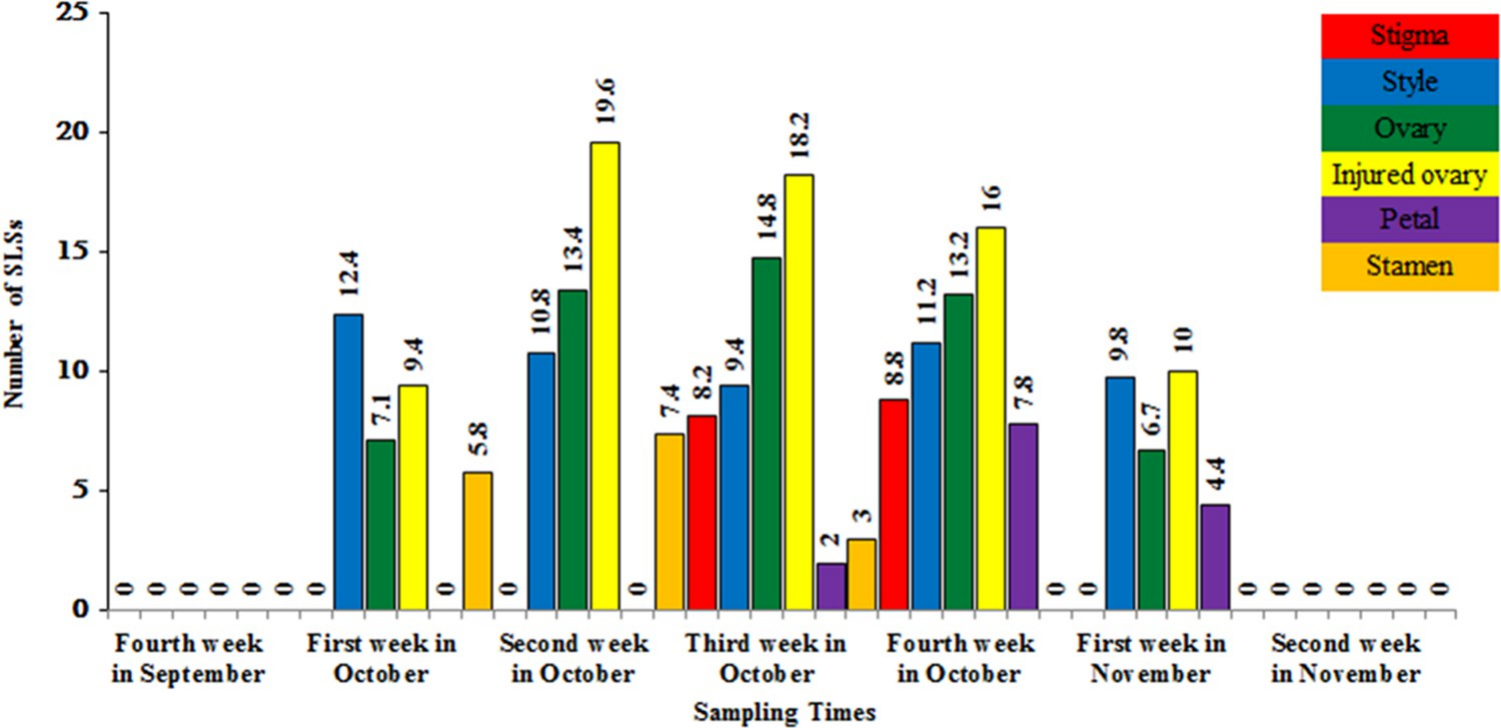
Effect of sampling time of saffron flower parts on the number of regenerated SLSs. The sampling times that not shown in this figure did not produce any SLS.

**Fig 3:**
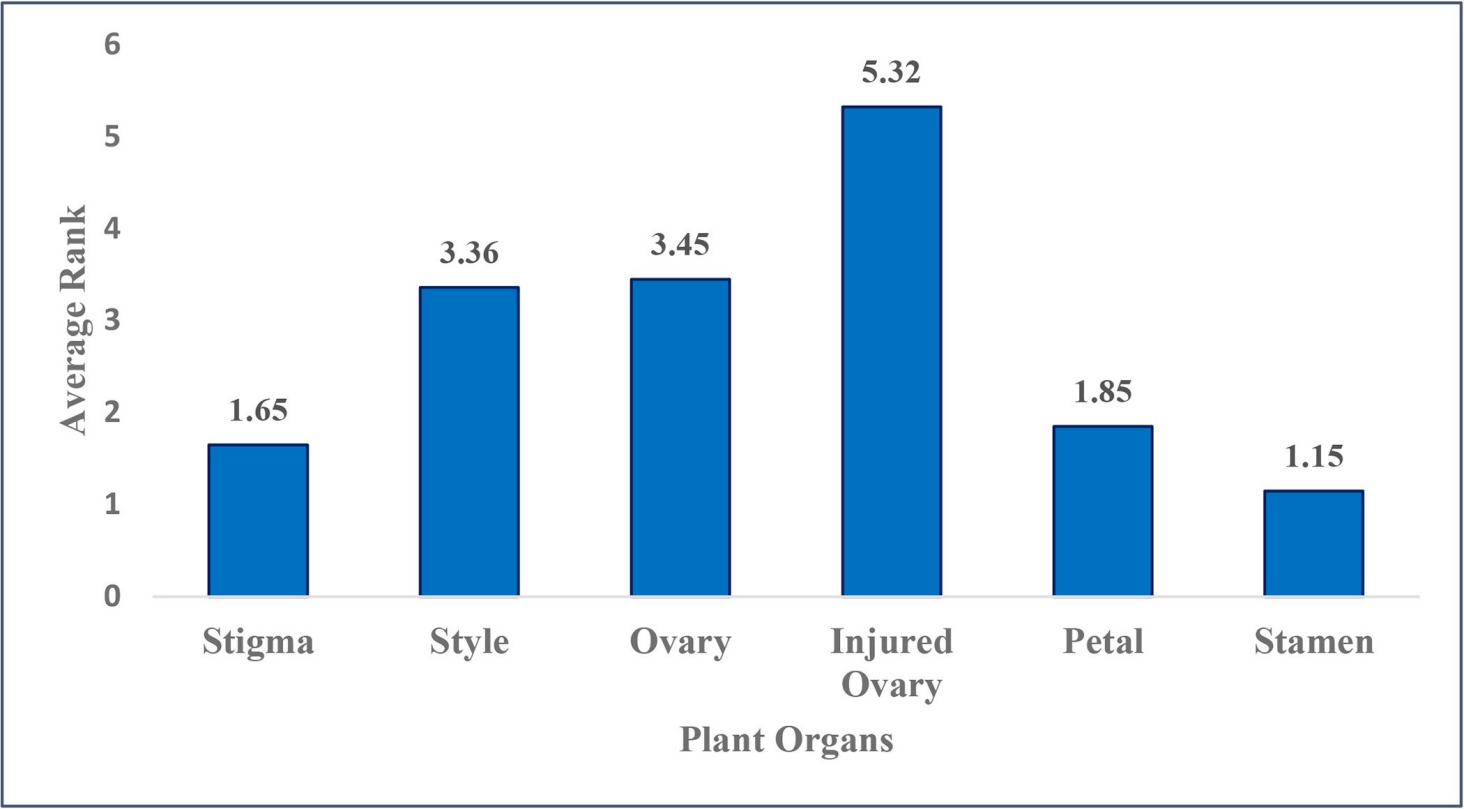
Average rank of the effects of saffron flower explants on *in vitro* SLSs formation.

The results of the first experiment, revealed distinct optimal sampling times for the various flower parts of saffron, including the stamens, stigma, style, ovary, and petal. Each flower part has a unique physiological age, which determines its ideal sampling time. Figs 2 and 3 illustrate the most suitable sampling time for each flower component. The ovary explants exhibited prolonged viability compared to other explants, indicating their superior responsiveness to hormone treatments. This extended viability contributes to a lengthened period of SLSs production, making ovaries a favorable choice for future studies. The data suggests that the third week of October is the overall optimal sampling time for all types of explants. However, it is essential to recognize that this timing is subject to variation based on geographical location and specific environmental conditions. The optimal physiological age for each explant may differ depending on flower type and environmental factors [39]. Our findings align with previous research [40–42], indicating that younger explants generally exhibit superior performance in SLSs formation. This study is the first to report the appropriate physiological age for each explant type based on their effectiveness in producing SLSs.

**Fig 4:**
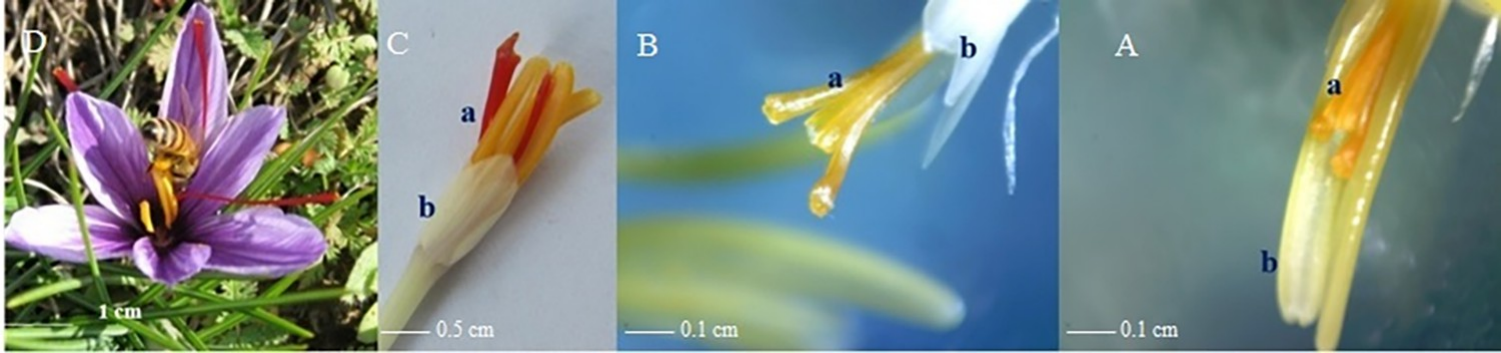
Saffron flower organs. (A): Comparison of stamens and stigmas in terms of physiological age and pigments amount in October. Stamens are completely yellow (a) and longer than the stigma, while the stigmas are not yet red and their size is shorter (b). (B): Petal and stigma comparison in terms of physiological age and pigment amount. Stigmas are still yellow (a), the petals are colorless and smaller (b), indicating the petals as the youngest part of the saffron flower. (C): Saffron flower at the beginning of November. Stigmas and stamens are mature (a), while the petals (b) remain colorless, small and younger; (D): A mature saffron flower in November. Stigmas are longer than the stamens and petals. Petals are purple and fully developed.

The results of this experiment substantiated the distinct capacities of flower explants in forming SLSs. The findings underscored that the physiological age and degree of differentiation of explants were the two essential factors influencing their *in vitro* responses. The younger flower parts demonstrated superior performance, attributed to the ongoing mitotic cell division in their meristematic tissues, a phenomenon further enhanced by the application of auxin and cytokinin [42]. Saffron exhibits protandrous flowering, with the ovary being younger than the stamens within the closed flower bud. This likely accounts for the more favorable response of the ovary and style compared to the stamen in the initial weeks of saffron flower differentiation, as observed in the first experiment. Different parts of the saffron flower undergo rapid organelle differentiation to coincide with the production and accumulation of colored pigments (Fig 4).

In this study, a detailed examination of flower buds before opening revealed the maturity of stigmas and stamens, while the petals of young flowers remained colorless. Notably, young petals and stamens exhibited responsiveness to certain treatments even before complete differentiation. SLSs were generated from the base of the young meristem, the point of connection between them. Additionally, in contrast to mature stigmas, the colorful end of the style produced SLSs under the influence of high concentrations of cytokinin and auxin.

#### The effects of PGRs on the *in vitro* formation of SLSs

**Fig 5:**
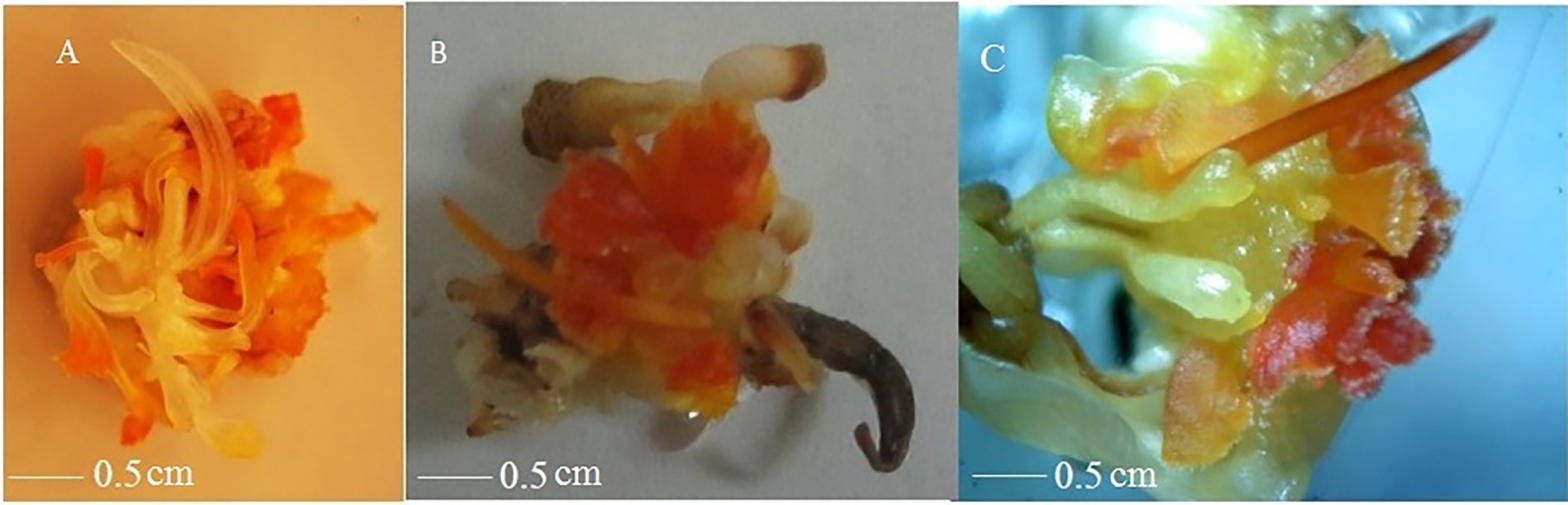
Saffron’s SLSs induction in different flower parts. Style (a), intact ovary (b), and injured ovary (c).

In the second experiment, the impact of common plant growth regulators, including auxins (NAA, IBA, and 2, 4-D) and cytokinins (BAP and Kin), on the induction of SLSs in saffron was investigated. The most effective induction of SLSs was observed in MS medium supplemented with 5 mg/L of NAA and 5 mg/L BAP and also, 10 mg/L of NAA and 10 mg/L Kin in injured ovaries, resulting in a success rate of 28%. Intact ovaries treated with the same hormone combination yielded a slightly lower induction rate of 27%. Table 2 shows the best hormonal compositions for each explant. Fig 5 shows SLSs induction in different explants.

**Table 2:** The best hormone combinations for each type of explant.

Explant type	Treatment code	Hormone combination (mg/L)	
Injured ovary	130	5 BAP	5 NAA
Injured ovary	36	10 Kin	10 NAA
Intact ovary	130	5 BAP	5 NAA
Intact ovary	36	10 Kin	10 NAA
Intact ovary	144	10 BAP	10 NAA
Intact ovary	143	10 BAP	7/5 NAA
Style	142	10BAP	5NAA
Style	130	5BAP	5NAA
Stigma	144	10BAP	10NAA
Stigma	22	5Kin	5 NAA
Stamen	144	10BAP	10NAA
Stamen	143	10BAP	7/5NAA
Petal	137	7/5BAP	7/5NAA
Petal	144	10BAP	10NAA

Hormone treatment results consistently demonstrated that the most successful SLSs induction across all flower parts was achieved with treatments containing NAA and BAP (Table 2). Similar hormone combinations have been reported for SLSs development on LS medium using half ovary explants [3], young ovary and half ovary explants [3, 42, 43], and floral bud explants [15, 17].

Substituting NAA with IBA or 2, 4-D significantly reduced the percentage of SLSs induction, highlighting the importance of NAA in the process. Among the various auxin and cytokinin combinations tested, the lowest SLSs induction rates were observed with different concentrations of IBA + Kin and 2, 4-D + Kin. Conversely, MS medium supplemented with NAA and BAP yielded the most favorable response, surpassing other auxin and cytokinin combinations and concentrations. These findings suggest that both the type and ratio of specific auxins and cytokinins are critical factors for successful SLS induction in saffron. Similar observations have been reported in other medicinal plants, such as *Calotropis gigantea* [44] and *C*. *sativus* [19, 45]. Furthermore, our study revealed that a concentration range of 5–10 mg/L NAA and 5–10 mg/L BAP is necessary for SLS formation in different saffron flower parts. Our research aligns with previous studies on the role of NAA and BAP in the formation of *in vitro* SLSs in saffron. For instance, Zeng *et al*. (2003) demonstrated that different concentrations of NAA and BAP can induce SLSs formation in saffron, and they also found that this combination can directly induce SLS from explants, bypassing callus formation [17]. Otsuka *et al*. (1992) discovered that using NAA and BAP in combination with alanine can induce SLSs in saffron [46]. Studies by Namera *et al*. (1987), Koyama *et al*. (1988), Lu *et al*. (1992), and Loskutov *et al*. (1999) have also explored the impact of different concentrations of auxin and cytokinin on SLSs formation in saffron flower explants, providing insight into the optimal concentrations needed to induce this process [12, 47–49].

Among the studied explants, stamens and stigmas showed the least success in SLS induction (Fig 3). The prolonged viability of ovary explants compared to other explants made them more suitable for SLS production, especially in response to plant growth regulators treatments. Similarly, Hosseinzadeh Namin *et al*. (2010) found that ovary explants had a longer viability compared to other explants and were more responsive [19].

#### The effects of culture media on the *in vitro* formation of SLSs

The results of the third experiment indicate that the B5 medium exhibited superior performance in SLSs production compared to the MS, 1/2 MS, and LS media, respectively. The results of mean comparison indicated that the B5 medium was the most suitable medium for inducing SLSs in most explants. In contrast, the LS medium was not a suitable, and in many treatments, the explants turned black and did not respond to hormonal treatments after being placed in the LS medium. For the explants of style and ovary, the B5 medium appeared to be the most suitable medium compared to the other three media. These results are along with the findings of other researchers who have also reported the superiority of B5 medium in SLS production [50]. The increased SLSs production in B5 medium is likely due to its favorable mineral and organic nutrients. B5 culture medium differs from MS culture medium in the amounts of macro- and micronutrients used. B5 culture medium contains higher amounts of (NH_4_)_2_SO_4_ and Ca (NO_3_)_2_·4H_2_O. Overall, the macronutrients content of B5 medium is lower than that of MS medium (4530 mg l^-1^ in MS and 3184 mg l^-1^ in B5). The micronutrients content is also higher in MS medium (103.33 mg l^-1^ in MS and 81.15 mg l^-1^ in B5). Additionally, B5 medium lacks glycine, but contains 10 times more nicotinic acid and pyridoxine, and 100 times more thiamine than MS medium. Our findings support the idea that a diverse array of basal media, concentrations, and combinations of various additives can effectively initiate and produce colored SLSs on half-ovary explants, aligning with prior studies [14, 43, 51].

In our study, different parts of saffron flower were utilized as explants for establishing SLSs formation. The significant impact of explant type on the organogenesis has been reported in various plants, including *C*. *sativus* [52], *Cnidium officinale* [53], and *Dendrocalamus latiflorus* [54], among others.

In general, it appears that the combination and ratio of auxin and cytokinin play crucial roles in determining the number and type of SLSs produced. The results indicate that as this ratio decreases, there is a reduction in callus formation, leading to direct SLSs formation. Conversely, an increase in this ratio resulting in indirect SLSs formation. Furthermore, it was observed that direct SLSs formation occurs exclusively in different parts of the pistil (ovary, style, and stigma), with no direct SLSs produced in any other part of saffron flower. It is noteworthy that direct SLSs, in terms of quality, are more favorable compared to indirect SLSs, on the other hand in terms of quantity, indirect SLSs demonstrate a greater ability for commercial-scale production.

The number of SLSs per explant in this study ranged from 0 to 24 (Fig 2). Kohda *et al*. (1993) observed a frequency of 16 or more SLSs per explant (half-ovary) after culture for 4 months [51], while Loskutov *et al*. (1999) observed 8–12 SLSs on B5 or LS medium supplemented with a wide range of levels of NAA or IBA and BA [49]. Additionally, Koyama *et al*. (1988) reported a frequency of 10–20 SLSs per explant (injured ovary) on LS medium [12]. Previous studies and our research findings indicate that SLSs can be produced from the stigma and ovary of saffron flowers. Our investigation, however, is the first to report SLSs induction from other flower explants, including the style, petal, and stamen.

### Flow cytometric analysis

Results from examining the ploidy level of the *in vitro* SLSs using flow cytometry showed no difference in the ploidy level of field stigmas and in vitro SLSs; all of them were triploid.

### HPLC analysis of the crocin, picrocrocin, and safranal

**Fig 6:**
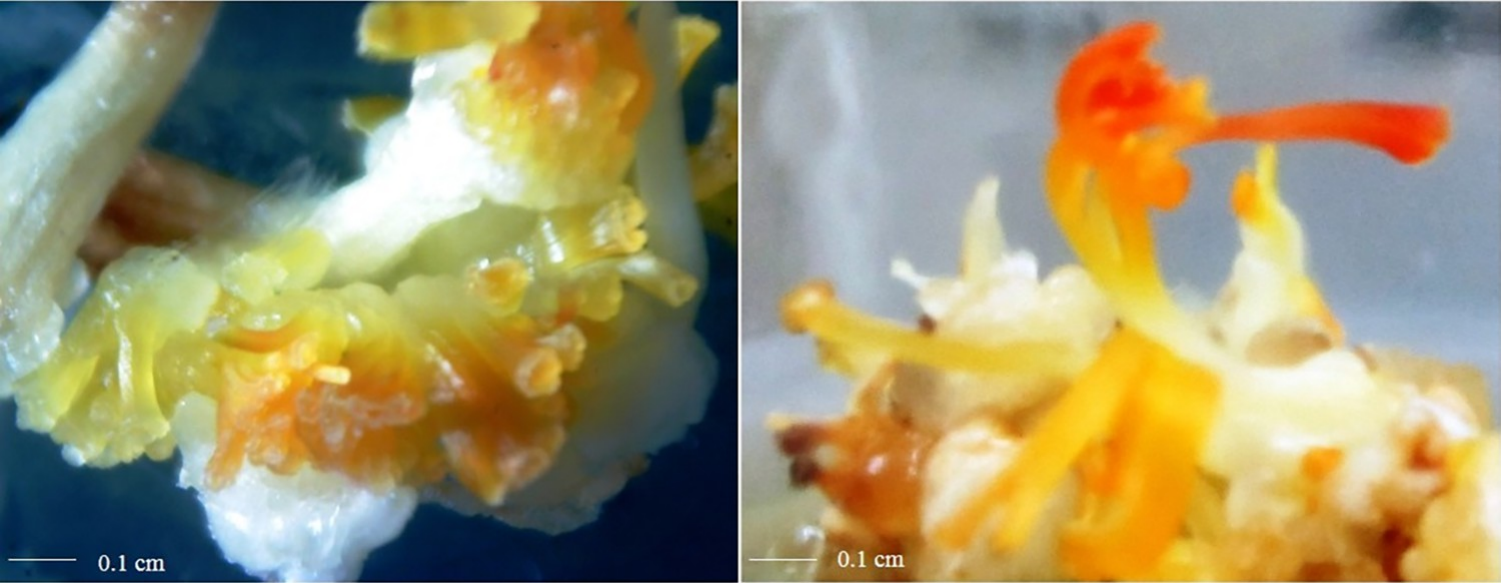
Saffron *in vitro* regenerated SLSs. Direct (right) and indirect (left) regeneration.

Based on our analysis, there were significant differences in the biochemical composition (crocin, picrocrocin, and safranal) between saffron *in vivo* stigma and *in vitro* SLSs. Table 3 presents the values of the detected compounds in the *in vitro* regenerated SLSs and in the *in vivo* stigma, along with previous reports on the same components in the SLSs and field stigma. The highest concentration of all three studied secondary metabolites was found in field-grown saffron. Among the *in vitro* regenerated SLSs, the SLSs directly regenerated on the style explant had the highest amount of the secondary metabolites (24.4 mg/g), followed by SLSs directly regenerated from stigma and ovary explants (23.8 and 21.4 mg/g, respectively). The lowest amount was observed in SLSs indirectly regenerated from ovary explant (19.3 mg/g). The results indicate that the SLSs obtained from flower style, had the highest accumulation of the studied secondary metabolites. Divergent reports exist regarding the production of apocarotenoids in SLSs, with some studies indicating lower apocarotenoid levels in SLSs compared to natural stigmas [3, 16] while others suggest similar production, under *in vitro* and *in vivo* conditions [11]. Interestingly, there is a report where a higher amount of crocin was found in SLSs compared to natural stigmas [49] (Table 3).Various studies have reported different quantities of important secondary metabolites in saffron *in vitro* SLSs. The differences in these reports may be attributed to factors such as the initial explant, and the type of SLS regeneration, whether direct or indirect. In our study, we selected the Saffron SLSs obtained through direct and indirect regeneration from the top three treatments for examination using HPLC. The SLSs regenerated directly were longer, fewer in number, and red, similar to *in vivo* saffron, while the indirectly regenerated SLSs were more numerous, shorter, and dark orange (Fig 6). In general, the indirect regeneration system produced a greater number of SLSs.

**Table 3:** Comparison of amount (%) secondary metabolites in dried tissue from field saffron stigmas and *in vitro* SLSs. Standard deviations are shown in the parentheses.

	Secondary metabolites (mg/g)				
SLSs / Field stigma	Crocin	Picrocrocin	Safranal	Method	Reference
SLSs from half ovary	5.9	21.9	0.013	HPLC	Loskutov *et al*., 1999 [49]
SLSs from half ovary	5.8	6.9	-	HPLC	"
SLSs from half ovary	0.2	0.03	0.005	HPLC	Himeno and Sano, 1987 [43]
Field stigma	14	3.6	0.039	HPLC	
SLSs from stigma	0.3	0.6	-	HPLC	Sarma *et al*., 1990 [42]
Field stigma	2.4	3.9	-	HPLC	
SLSs from half ovary	0.13	14.2	0.480	Thin Layer Chromatography (TCL), spectrophotometry, Chemical methods	Visvanath *et al*., 1990 [62]
Colored callus	0.05	18.2	1.2		
Field stigma	1.7	3.8	1.44		
SLSs from half ovary	low level of apocarotenoids compared with field stigma			Spectrophotometery	Mir *et al* 2015 [3]
**Direct regenerated SLSs from style**	**10.2** ^**b**^ **(±0.65)**	**4.5** ^**c**^ **(±0.2)**	**9.7** ^**b**^ **(±0.11)**	**HPLC**	**Present study**
**Direct regenerated SLSs from stigma**	**9.8** ^**b**^ **(±0.26)**	**4.8** ^**b**^ **(±0.17)**	**9.2** ^**c**^ **(±0.11)**	**HPLC**	
**Direct regenerated SLSs from ovary**	**8.5**^**c**^ **(±0.36)**	**4.2** ^**d**^ **(±0.1)**	**8.7** ^**d**^ **(±0.17)**	**HPLC**	
**Indirect regenerated SLSs from style**	**8.2** ^**c**^ **(±0.26)**	**3.3** ^**e**^ **(±0.05)**	**7.7** ^**e**^ **(±0.15)**	**HPLC**	
**Indirect regenerated SLSs from stigma**	**8.1** ^**c**^ **(±0.20)**	**2.8** ^**f**^ **(±0. 1)**	**7.5** ^**e**^ **(±0.41)**	**HPLC**	
**Indirect SLSs from ovary**	**7.2** ^**d**^ **(±0.20)**	**4.5 (±0.02)** ^**c**^	**7.6** ^**e**^ **(±0.17)**	**HPLC**	
**Field stigma after flowering**	**15.25** ^**a**^ **(±0.31)**	**5.78** ^**a**^ **(±0.04)**	**13.28** ^**a**^ **(±0.11)**	**HPLC**	

The direct regenerated SLSs displayed a biochemical profile more closely resembling that of field saffron stigmas compared to the indirect ones. Compared to direct SLS regeneration, the indirect regeneration through the callus stage seems to induce more changes in the regenerated SLS. This is likely due to the genetic instability induced during the callogenesis phase. The callogenesis involves dynamic changes in cells, potentially leading to significant differences compared to their original state [55]. This crucial period can provide insights into the variations observed between indirectly and directly regenerated SLS. The callus phase may act as a catalyst for genetic and epigenetic modifications, ultimately resulting in heightened genetic instability. Subculturing the calli could further amplify this instability due to the accumulation of mutations or chromosomal alterations over time. These findings align with other studies suggesting that callogenesis plays a regulatory role in the expression of genes involved in the biosynthetic pathways of secondary metabolites [56].

It’s important to compare field stigma and *in vitro* SLSs in a proper manner. Since metabolite accumulation in the field increases with development, it is essential to consider the developmental stage when comparing the measured metabolites in the field stigma and *in vitro* SLSs. Notably, the amount of stigma carotenoids increases before the flowers open, reaching its peak. Subsequently, after the flower opens, the amount of these metabolites decreases, and environmental conditions such as high temperature (40°C) and relative humidity (75%) can further affect the color, aroma and flavor of saffron [57, 58]. Therefore, sampling immediately after flowering represents the optimal time for measuring important metabolites. Differences in the observed values in field stigmas (Table 3) may be due to the sample’s origin, different drying processes with varying time periods, and storage conditions in each country [31]. These factors can influence the concentration of glycosidic carotenoids due to their thermal sensitivity and susceptibility to light [59]. The observed reduction or lack of response in relatively older or advanced stage explants may result from qualitative and quantitative changes in DNA, cell cycle status, endogenous hormonal levels, and genotype [60]. One of the reasons for the notable differences in metabolites amounts in this report appears to be the attention to explant type as well as the consideration of the type of SLS regeneration (direct or indirect). Based on the previous reports various factors can increase the production of secondary metabolites in saffron tissue culture including sucrose, iron, copper, potassium, gibberellin, polyamines, and a combination of sucrose and iron [61]. Moreover, elicitors can be employed as an effective tool to enhance the production of secondary metabolites in saffron. Recently, studies have shown that salicylic acid (SA), polyethylene glycol (PEG), and ultrasonic waves can be used as effective elicitors to increase the production of crocin and safranal in saffron suspension cultures. These elicitors stimulate the expression of genes controlling crocin and safranal biosynthesis and increase the activity of related enzymes, leading to an increase in the production of these secondary metabolites. Treatment with salicylic acid and polyethylene glycol increased crocin and safranal production. The use of elicitors can be a viable approach to enhance the production of secondary metabolites in saffron and can contribute to the development of new methods for producing these valuable metabolites [61].

## Conclusion

In the present study, we investigated the production of *in vitro* SLSs as an alternative method for saffron stigma production. The B5 medium, enriched with 5–10 mg/ L BAP and 5–10 mg/ L NAA was the most effective treatment for inducing SLSs in all types of explants. The intact, and injury ovary explants were found to be the most effective explant types for SLSs formation. The highest accumulation of the three studied secondary metabolites was observed in the SLSs of style, stigma, and ovary explants, respectively. A biochemical comparison of regenerated SLSs with field stigmas revealed that the *in vitro* SLSs can produce the most important secondary metabolites of saffron, with concentrations similar to those of field stigma. Besides the type of explant, the method of regeneration can also impact the number of SlSs and the concentration of secondary metabolites. Therefore, choosing the appropriate explant and SlSs regeneration method is crucial for optimizing SLSs regeneration as an alternative method for producing secondary metabolites of saffron.

This study found that the highest concentrations of metabolites were obtained in the directly regenerated SLSs. However, it also demonstrated that these SLSs can be used as explants to produce new SLSs. If the method is optimized to produce the maximum number of SLSs per explant, the yield of metabolites can be increased through successive subcultures.

The amounts of three metabolites examined in indirectly regenerated SLSs were slightly less than those of the directly regenerated SLSs. However, the indirect regeneration system has the potential for mass production of SLSs, thereby increasing the production of metabolites. Therefore, it is necessary to optimize the conditions for mass production of callus from desirable explants and conduct research to produce the maximum number of SLSs on the calli. Additionally, it is essential to study the effects of different elicitors to increase the biosynthesis of the mentioned metabolites in the SLSs.

## Recommendations

This study highlights the potential of *in vitro* production of SLSs as an alternative method for producing saffron stigma. The findings indicate that the optimal sampling time for most explants is the third week of October. Other researchers are encouraged to re-examine this timing based on their specific geographical regions.

## Future perspectives

Biochemical analysis indicated that SLSs regenerated directly from the style explants produced the highest concentrations of, picrocrocin, and safranal, closely resembling the quality of field-grown saffron stigmas. Although the indirect regeneration system produces slightly lower metabolite concentrations, it has significant potential for mass production. It is essential to balance the quality and quantity of SLSs produced. Future research should optimize *in vitro* conditions for direct and indirect regeneration to maximize the yield of SLSs and secondary metabolites. More detailed studies are recommended to refine various factors including modifications to the B5 culture medium. This may involve investigating zeatin and thidiazuron and adjusting the levels of macroelements and microelements. Additionally, it is important to consider incubating conditions, such as the temperature and container types, along with subculturing techniques to ensure a consistent, high-quality product. Utilizing precursors and elicitors may also enhance the production of the targeted secondary metabolites, making *in vitro* production methods more viable for commercial applications.

## Supporting information

S1 Table The hormonal composition’s effects on the number of SLSs produced in the intact ovary. (DOCX)

S2 Table The hormonal composition’s effects on the number of SLSs produced in the injured ovary. (DOCX)
