# Supplementary material for: Induction of stigma-like structures in saffron (Crocus sativus L.): Exploring factors and metabolite analysis
Source: PLoS One. 2025 Jan 13;20(1):e0317186. doi: 10.1371/journal.pone.0317186 (PMC11730422; doi:10.1371/journal.pone.0317186)
Supplement: S1 Table — (DOCX) [file pone.0317186.s001.docx]

Table S1. The hormonal composition's effects on the number of SLSs produced in the intact ovary.

| **Treatment code** | **Hormonal composition (mg.l^-1^)** | | | | | **Number of SLSs/i**ntact ovary |
| --- | --- | --- | --- | --- | --- | --- |
|  | **BAP** | **Kin** | **NAA** | **IBA** | **2,4-D** |  |
| 130 | 5 | - | 5 | - | - | 25 |
| 36 | - | 10 | 10 | - | - | 21 |
| 144 | 10 | - | 10 | - | - | 19 |
| 143 | 10 | - | 7.5 | - | - | 18 |
| 142 | 10 | - | 5 | - | - | 17 |
| 137 | 7.5 | - | 7.5 | - | - | 12 |
| 136 | 7.5 | - | 5 | - | - | 12 |
| 168 | 5 | - | - | 10 | - | 11 |
| 29 | - | 7.5 | 7.5 | - | - | 10 |
| 22 | - | 5 | 5 | - | - | 9 |
| 28 | - | 7.5 | 5 | - | - | 9 |
| 30 | - | 7.5 | 10 | - | - | 8 |
| 214 | 10 | - | - | - | 5 | 8 |
| 65 | - | 7.5 | - | 7.5 | - | 8 |
| 213 | 10 | - | - | - | 2.5 | 6 |
| 209 | 7.5 | - | - | - | 7.5 | 6 |
| 212 | 10 | - | - | - | 1 | 6 |
| 159 | 2.5 | - | - | 2.5 | - | 6 |
| 15 | - | 2.5 | 2.5 | - | - | 5 |
