# Supplementary material for: Induction of stigma-like structures in saffron (Crocus sativus L.): Exploring factors and metabolite analysis
Source: PLoS One. 2025 Jan 13;20(1):e0317186. doi: 10.1371/journal.pone.0317186 (PMC11730422; doi:10.1371/journal.pone.0317186)
Supplement: S2 Table — (DOCX) [file pone.0317186.s002.docx]

**Table S2.** The hormonal composition's effects on the number of SLSs produced in the injured ovary.

| **Treatment code** | **Hormonal composition (mg.l^-1^)** | | | | | **Number of SLSs/injured ovary** |
| --- | --- | --- | --- | --- | --- | --- |
|  | **BAP** | **Kin** | **NAA** | **IBA** | **2,4-D** |  |
| 130 | 5 | - | 5 | - | - | 28 |
| 36 | - | 10 | 10 | - | - | 25 |
| 144 | 10 | - | 10 | - | - | 18 |
| 142 | 10 | - | 5 | - | - | 18 |
| 143 | 10 | - | 7.5 | - | - | 17 |
| 168 | 5 | - | - | 10 | - | 12 |
| 28 | - | 7.5 | 5 | - | - | 12 |
| 29 | - | 7.5 | 7.5 | - | - | 11 |
| 22 | - | 5 | 5 | - | - | 11 |
| 137 | 7.5 | - | 7.5 | - | - | 10 |
| 136 | 7.5 | - | 5 | - | - | 8 |
| 30 | - | 7.5 | 10 | - | - | 8 |
| 65 | - | 7.5 | - | 7.5 | - | 7 |
| 15 | - | 2.5 | 2.5 | - | - | 6 |
| 214 | 10 | - | - | - | 5 | 5 |
| 213 | 10 | - | - | - | 2.5 | 5 |
| 14 | - | 2.5 | 1 | - | - | 5 |
| 101 | - | 7.5 | - | - | 7.5 | 5 |
| 135 | 7.5 | - | 2.5 | - | - | 5 |
